# Telehealth and chronic pain management from rapid adaptation to long-term implementation in pain medicine: A narrative review

**DOI:** 10.1097/PR9.0000000000000912

**Published:** 2021-03-09

**Authors:** Jordi Perez, Kacper Niburski, Michelle Stoopler, Pablo Ingelmo

**Affiliations:** a Alan Edwards Pain Management Unit and Cancer Pain Clinic, McGill University Health Centre, Montreal, QC, Canada; b Alan Edwards Centre for Research on Pain, McGill University, Montreal, QC, Canada; c Faculty of Medicine, McGill University, Montreal, QC, Canada; d Chronic Pain Service, Montreal Children's Hospital, McGill University Health Centre, Montreal, QC, Canada

**Keywords:** Telemedicine, Remote consultation, Chronic pain

## Abstract

The COVID-19 pandemic called for drastic changes to expand and rapidly implement telehealth to prevent breach of care for chronic patients. Responding to the challenge of implementing remote care in chronic pain services, a specialty highly dependent on doctor–patient rapport, physical examination, and frequent follow-up visits requires extensive adaptation involving administrative processes and clinical routines. We present our experience of a successful rapid adaptation to telemedicine paradigm as a response to the COVID-19 pandemic during a time of marked restriction of access to ambulatory hospital services for pediatric and adult chronic pain patients. This narrative review covers current scientific evidence for the use of telehealth for chronic pain management and describes in detail the challenges to implement telemedicine in ambulatory clinics from different perspectives. Best practices for telehealth use are recommended. A proposal for remote physical examination of pain patients is made, based on available evidence in the fields of musculoskeletal medicine and neurology comparing in-person vs remote physical examination. As an internal quality control process, an informal online survey was conducted to assess thoughts and experiences among patients and caregivers using telemedicine consultation services at the pediatric pain clinic. Providing chronic pain management services through telehealth is a viable option for many patients and health care professionals. This is reliant on the availability of appropriate materials and training, with guidelines for both patients and health care workers. With the rapid pace of technological advancements, even further integration of telehealth into routine health care is possible.

## Abstract 2

Telehealth services for chronic pain management are a vast new field with many potential applications for providing high quality, patient-centered care.

## 1. Introduction

The modern era of telehealth (TH) started in the late 1960s when the Massachusetts General Hospital introduced the first hospital-based, multispecialty telemedicine practice by offering remote clinical examinations to travelers and workers at Logan International Airport.^39^ Since then, TH has emerged as an alternative tool to provide both acute and chronic care to patients with difficulty accessing health care facilities.

Under the umbrella of TH, different concepts of providing remote, high quality clinical care to protect the patient and the clinician all involve information and technology systems. Moving clinical care closer to the patient by providing remote yet expedite assessment and treatment, facilitating clinician-to-clinician case consultation, and avoiding unnecessary travel to health care facilities, are some of the most important TH goals.

In the last 2 decades, the overall clinical benefits of TH approaches compared with in-person care have been extensively reviewed. The most convincing evidence comes from teleradiology, telemental health care, transmission of echocardiographic images, teledermatology, and home telecare. Positive findings included savings and clinical benefits through avoidance of travel and associated delays.^15^ Telehealth cost-effectively reduces use of hospitals and improves patient compliance, satisfaction, and quality of life. Patients accessing TH-supported health services feel more confident and empowered, with better knowledge and improved health outcomes, and experience better nurse–patient relationships.^11^ Barriers to greater implementation of TH services include lack of evidence-based research on long-term outcomes and unforeseen harm or consequences,^12^ potential inequity in access, and use in populations such as elderly and disabled patients.

The American Telemedicine Association^37^ has suggested a set of guidelines to support the practice and growth of telemedicine. In addition, professional regulatory bodies have designed their own TH directives to be applied regionally.^34^

## 2. Telehealth for chronic pain management, current scientific evidence

Remote assessment has mainly focused on nurse-led phone services to monitor treatments performed in-person at pain centers or to quickly respond to patients' concerns. This has been demonstrated to be feasible and effective when monitoring postoperative,^35^ pediatric,^23^ chronic cancer,^33^ and noncancer^5^ pain patients and providing treatment for chronic pain. Nonmedical self-management strategies such as psychology^30^ or physical-based approaches^1^ delivered by phone^17^ or internet-based are the uses with better evidence.^10^

## 3. Use of telehealth during the COVID pandemic

The onset of the COVID-19 pandemic triggered an urgent need to safeguard continuity of care for patients during hospital closures of ambulatory units and nonurgent in-hospital consultations and enabled compliance with new requirements for social distancing. Scientific pain societies, such as the International Association for the Study of Pain^10^ and jointly the American and European Associations of Regional Anesthesia and Pain Medicine,^29^ recommended implementation and integration of telemedicine consults into routine clinical care. Urgent implementation recommendations left little time for design.

### 3.1. The telehealth approach to chronic pain patients at McGill University Health Centre

The McGill University Health Center serves the population of a very large geographical area in the province of Quebec, stretching from Montreal close to the USA border to Nunavik in the far North, comprising approximately 1.9 million people from different communities and all walks of life.

A dedicated TH service was begun in 2012 to provide super-specialized health care closer to patients' homes. This service connected patients, local clinical teams, and specialized care clinics in tertiary hospitals. Its success relied on the quality of the communication which included a dedicated technician, monitors and video cameras, and the use of sophisticated videoconference software (IRIS) connecting the patient's local health center and our university center. This high-quality and costly remote consultation option was offered mostly to the population of the far North and included chronic pain consultations among its menu of services.

McGill University Health Center has 4 dedicated pain units (adult chronic noncancer pain clinic, an adult cancer pain clinic, a pediatric chronic pain service, and a dedicated neuromodulation and neuropathic pain unit) with an estimated activity of more than 17,500 consultations per year. Prepandemic, remote pain services were limited and included the following: clinical video consultation using the McGill Telehealth services as described above and remote symptom management through nurse-led phone consultation services.^19,24^ In September 2019, direct patient consultations through video call with ZOOM for pediatric pain cases were added.

All nonurgent ambulatory visits were cancelled once the pandemic began. An internal audit comparing January 15th with March 15th of 2019 with the same period in 2020 comparing the number of consults between revealed a significant decrease in in-person new patient consultations and treatments and an increased number of phone consultations. The total number of follow-up visits at different clinics increased by 30% to 300% (internal results, data nonpublished). The pediatric pain clinic had already integrated the video call visit as an optional service a few months before the pandemic. During the first wave of COVID-19 in Canada, the pediatric pain clinic completely switched all appointments to video calls with only needing a ramp up of the existing service.

For the adult pain population, all visits were turned into phone assessments immediately, and it took 2 weeks to develop the necessary processes to establish TH services. Then, the new protocol mandated all follow-up consultations conducted preferably by phone although video was available on request, and new patient assessments by video call unless otherwise indicated by the treating clinician. Amid the second wave, hospital directives mandate no more than 50% visits in person and these protocols allow ample observance of those rules; in addition, the fear of contracting an infection by coming to a health care facility makes this option particularly appealing to vulnerable patients such as those diagnosed with cancer, poor mobility, and/or with comorbidities.

### 3.2. Adapting to remote chronic pain care

The transition to a low-budget telemedicine system, independent from an IT department, requires major changes to administrative teams, equipment, patient routines, and clinician practices.

### 3.3. Administrative changes

Never sufficiently acknowledged is the crucial responsibility of administrative teams in the remote care of the pain patient. Consultations shifted to remote require individual phone calls to each patient to set the date of the remote visit, clarify the method of communication, and, most importantly, remind them not to come to the hospital. Newer routines such as recommending patients to wait for the remote consultation at a quiet and private location had to be instituted. Special attention must be paid to updating patients' contact details (eg, valid phone number/email address).

**Figure 1.**
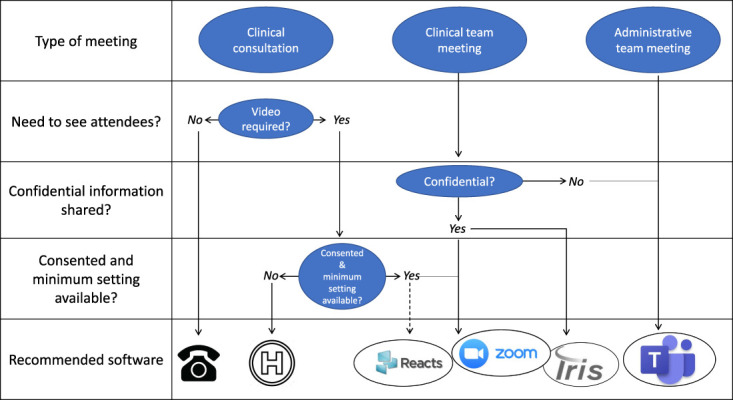
Algorithm proposal by the McGill University Health Centre to choose among the different platforms to conduct virtual meetings. REACTS, ZOOM, IRIS, and Microsoft TEAMS logos obtained from their websites.

The Quebec provincial government offered ZOOM, TEAMS, REACTS, and IRIS to health care providers. These platforms share safeguards for protecting patient confidentiality, including encrypted connections, password-protected meetings, disabling recording functions, and multiconference meetings, where the host controls attendance by a virtual waiting room. Figure 1 depicts an algorithm to help choosing between alternative platforms depending on the needs. Many clinicians were familiar with ZOOM from previous personal and professional experiences, and those interested in TH received a free professional ZOOM individualized license purchased by our state health authority valid for 1 year. These licenses additionally grant scheduling permission to designated administrative staff members on behalf of clinicians.

Arranging a video conference with a patient requires multiple time-consuming steps. First, patients must be comfortable with the concept of a remote consult and must have the appropriate equipment available. Patients with limited technology skills need training to download and install the software and test it before the visit. Our provincial health authorities created online educational videos for patients, but often, this time-consuming training is instead provided by the secretary scheduling the video call, sometimes challenging the wits, and patience of both patients and staff.

The actual scheduling of a video call is best performed through an e-mail message that contains a direct link to the meeting. Emailing patients must follow strict ethical rules such as using a secure server and a corporate e-mail account. Readers from busy and understaffed government-funded pain clinics will appreciate the difficulty of accessing precious and scarce resources needed to increase administrative staff to e-mail patients. Alternatively, patients can download the software and wait for a phone call to get the meeting details (ID and passcode) minutes before the consult. The clinician and/or staff can assist the patient through the login steps by telephone, when required. Occasionally, the image will come through but not the sound. In those scenarios, after confirming the patient's microphone is not set on mute, the encounter can be performed by combined telephone for sound and video for image (author's personal experience).

### 3.4. Tools for remote visits

The tools required for remote consultation have long been available, but public hospitals may not be able to afford the cost of updating their equipment, especially with the financial stress on government agencies during a deadly pandemic. Telephone consultations require sets with speakers or with headsets to allow hands-free calls. For teaching purposes, telephones with speakers allow trainees to hear both sides of the conversation. These options are easily found at secretarial facilities but rarely in medical offices. In our experience, rapid equipment acquisition requests were not always feasible.

Video conferences require a sufficient internet bandwidth, video, speakers, and microphones. Many hospital computers are connected to the Internet by landlines but, rarely, have integrated cameras and microphones. Laptops, when available, are probably better, but relying on wireless internet connections of adequate bandwidth can present a challenge. In the midst of the COVID-19 pandemic, it was certainly difficult, if not unrealistic, to expect that public institutions would prioritize purchase of new phone settings, headsets, webcams with integrated microphones, and/or updating their IT tools.

On the patients' side of the virtual connection, similar concerns apply about access to an adequate internet connection (minimum 10 Mb/sec). Especially during the pandemic, other household members may also be using the internet connection for teleworking or teleschooling.

The choice between using a smartphone or computer to receive clinical video calls merits a small analysis of its pros and cons, as depicted in Table 1. Although most of the studies on telemedicine have focused on desktop or web-based applications, the advent of mobile communication provides yet another avenue for patient–physician interactions. Although a simple telephone connection can provide an understanding of history, it is not enough; video is suggested as a mainstay of not only direct medical care but of providing an understanding of the social and behavioral relations.

**Table 1:** Comparison of different devices to conduct a video call.

	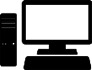 PC	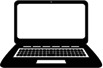 Laptop	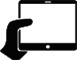 Tablet	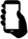 Smartphone
Quality of Internet connection	Landline connection (good)	Landline connection (rarely and good) Wi-Fi connection (often and variable)	Wi-Fi connection (always and variable) 3G connection (sometimes and good)	Wi-Fi connection (always and variable) 3G connection (often and good)
Image/sound tools	Not always available	Usually available	Available by default	Available by default
Portable	Not	Somewhat	Very much	Very much
Image of the clinician	Good and large	Good and large	Good and large	Good and small
Image of the patient	Good and stable	Good and stable	Good but may be unstable	Fair but often unstable

For clinical purposes, a device that can be placed or manipulated at different angles to facilitate the clinician's inspection of the patient is also advisable; regrettably, manipulation of these requires some technical competence. The learning curve might be steep until the technology itself becomes more standard in pain management.

### 3.5. Clinician considerations

Clinicians must abide by the specific rules set out by their respective professional authorities regarding remote clinical care. The rules and other directives necessary for the successful and compliant practice of telemedicine fall outside the scope of this review. They were widely available through articles, webinars, and continuing education activities before the COVID-19 pandemic began.

Whether patients are contacted by phone or video call, clinicians must document the patient's verbal consent to conduct the visit remotely. Patients must be aware and consent that remote visits bear limitations, making them potentially, less accurate compared with conventional in-person visit. In an ideal setting, the first consultation with the pain clinic should be conducted in-person, and the possibility of subsequent follow-up visits being performed remotely should be discussed and consented to in writing.

By telephone, a clinical interview is clearly feasible. However, the patients' body language cannot be assessed, and performing a physical examination is obviously impossible. It might be advisable to limit phone consults solely to patients already known to the clinician, with the understanding that subsequent video calls or in-person visits might need to be arranged at the discretion of the treating clinician.

Clinicians can conduct remote consultations from anywhere (hospital, clinic, and home) provided that they have secure access to the patient's chart. The practice of telemedicine in times when ambulatory settings should not be overcrowded facilitates interdisciplinary, concurrent evaluations by different team members working from different sites. Communication between team members must abide to the rules of patient confidentiality; therefore, personal methods of communication (personal e-mail, text messages, etc.) are discouraged by professional health bodies unless basic rules to protect patient's identity are observed.

Initial video consultation visits usually take longer, accounting for potential technical problems arising. Patients often rely on clinicians to solve potential glitches with the software. Recommendations include knowing basic troubleshooting for these malfunctions and being prepared with an alternative method of communication available for patients in case that trouble arises.

The etiquette for a video interview merits some thought. Whether clinicians are working from a desk at a health care institution or from home, professionalism should never be sacrificed for comfort. Physicians must introduce themselves and show their ID to the camera to allow patients to ascertain their identity. Similarly, patients must be identified by asking key personal questions only available in their chart, and when video is used, requesting patients to show a government-issued photo ID is also recommended.

Patients generally like being approached in the comfort of their own home, but this should never undervalue the professional relevance of the clinical encounter. Avoiding unnecessary distractions and dedicating the adequate attention to the clinician is a part of a respectful clinical interaction. Home and hospital or clinic settings might be noisy places at times, if background noises cannot be avoided, the use of a headset with integrated microphone significantly improves the voice transmission and reception of sound.

Most clinicians type their notes into electronic medical records nowadays. Sharing the video call and the note taking might cause some inconveniences if using small screens such as laptops or tablets. An alternative is using different devices for the video call and for accessing the electronic medical record. When this is the alternative, it must be acknowledged that patients may notice the clinician looking away from the camera. Because the lack of physician–patient eye contact can be a source of frustration and mistrust,^16^ this must be contemplated and acknowledged. Positioning the camera as close as possible to the computer could certainly provide reassurance to patients that they are being listened to and assist in maintaining the therapeutic relationship.

### 3.6. Clinician considerations: remote physical examination

**Figure 2.**
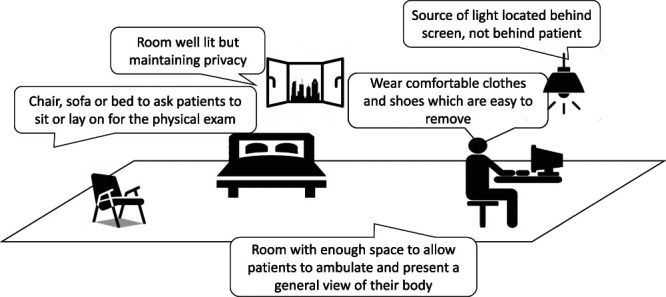
Ideal setting to perform a remote physical examination by video call.

The physical examination is a crucial part of the pain assessment for different clinicians. A summary is provided in Table 2. Of the various elements of a physical examination, namely, inspection, palpation, auscultation, percussion, and neurological and musculoskeletal assessment, inspection is by far the easiest to perform remotely and might yield the best results. Conversely, auscultation and percussion might be challenging. A virtual physical examination can never replace the more complete in-person assessment; nevertheless, it can help in building a working diagnosis. Recommended settings for performing a virtual physical examination are depicted in Figure 2. The final goal is to be able to achieve an optimal inspection of the patient and the pain syndrome. Patients should wear comfortable clothes and shoes that can easily be removed and allow for adequate movement. Undressing in front of a camera can be challenging and embarrassing for some patients. Clinicians must reassure them that the video call cannot be recorded with government-provided software and remind patients to keep it private by closing their window curtains. Patients should be at a well-lit room with enough space to allow distancing themselves from the camera to facilitate a distant view of their body habitus, posture, and their movements, including their gait. For detailed inspection, smartphones and tablets are preferred because they can be moved and pointed at specific anatomical areas. If laptops or computers are used, some anatomical areas may be more challenging unless their web camera can be detached.

**Table 2:** Examples or remote physical examination findings and testing that could corroborate a clinical impression (comprehensive list of tests not provided).

	Low back pain syndromes	Cervical pain syndromes	Upper-limb pain syndromes	Lower-limb pain syndromes	Head and face pain syndromes	Abdominopelvic pain syndromes
Inspection	Kyphotic or scoliotic habit Antalgic gait/posture Asymmetries	Kyphotic/lordotic habit Antalgic posture Asymmetries	Asymmetries Color, swelling changes	Asymmetries Color, swelling changes	Asymmetries Color, swelling changes	Scars Asymmetries Guarding
Palpation	Tight band Trigger point Mass	Tight band Trigger point Mass	Tight bands Trigger points Mass Joint swelling	Tight bands Trigger points Mass Joint swelling	Tight bands Trigger points Mass	Tight bands Trigger points Mass
Auscultation	Irrelevant	Irrelevant	Irrelevant	Irrelevant	Irrelevant	Desirable but not feasible
Percussion	Desirable but not feasible	Irrelevant	Irrelevant	Irrelevant	Irrelevant	Desirable but not feasible
NRL examination	Active straight leg raise test Tripod test Dermatome sensory test Myotome strength test	Spurling test Cervical tendinopathy Dermatome sensory test Myotome strength test	Tinel's sign Phalen's sign Peripheral nerve exam (sensory and motor)	Tinel's sign Tarsal -tunnel Weaknesses Heel-to-shin	Cranial nerves (III, IV, V, VI, VII, X, XI & XII) Facial twitch Weaknesses Neurovegetative signs.	Peripheral nerve sensory deficits
MSK examination	SIJ (FADIR, FABERE)	Active range of cervical flexion, extension, side bending, retraction/protraction	Active range of joint movement Bursitis, tendinitis	Active range of joint movement Bursitis, tendinitis	Active range of temporomandibular joint motion	Rebound Carnett test

Inspection alone can suggest diagnosis of certain pain syndromes. Examples include a scoliotic posture, an antalgic gait, a unilateral swelling of a limb, or neurovegetative changes accompanying a headache. Palpation is challenging through TH, yet patients can often tell if a certain anatomical area is particularly tender or tight to superficial or deep self-palpation. Presence of trigger points can be assessed by asking patients or a proxy to press those tender spots with enough force to turn their nail bed white, which is roughly estimated to equal 4 kg.^4^ Allodynia and hyperalgesia can be self-tested, albeit with challenges; household objects such as makeup brushes, Q tips, safety pins, ice cubes, and mugs with hot beverage can substitute for calibrated quantitative sensory testing utensils.

Some tests of the neurological^9^ and musculoskeletal clinical examination can be performed remotely by self-examination or assisted by a proxy. Research involving patients undergoing a remote physical examination with or without the assistance of advance measurement software have been compared with conventional physical examinations performed in person. This research focused on telerehabilitation of the shoulder,^31^ knee,^25^ and cervical^21^ body areas and demonstrated a significant degree of agreement between remote and in-person examinations. Calculation of validity, inter-rater and intrarated agreement yield consistently positive results suggesting that remote physical examination is a valid alternative. Authors unanimously conclude that there is value in remotely inspecting and assessing position and range of movements in patients with musculoskeletal disorders with pain; however, remote physical examination has limitations and cannot replace a conventional in-person examination.

In the fields of neurology and neurosurgery, basic recommendations for remotely conducting a neurological examination have been published.^6^ A direct comparison remote vs in-person has not yet been conducted. Interestingly, remote observation of a physical examination performed by a proxy (junior neurology resident) was compared with in-person examination, and results also showed a positive degree of correlation in certain tests such as facial strength, sensation, gait, and others.^9^ The concept-assisted physical examination with a proxy is challenging. It is reasonable to do so when the assistant has basic anatomical knowledge and sufficient hindsight to help with some passive movements, palpation, or sensory testing without reaching painful limits and causing unnecessary pain. Further research is needed to focus on the signs and symptoms that could be identified remotely that should trigger an urgent consultation with emergency symptoms in pain patients such as those with neurological spine or cancer pathology.

Auscultation is not commonly performed for a pain-oriented physical examination; however, it can be incorporated with the help of devices designed for that purpose. Examples include the application real-time mobile device heart teleauscultation that patients themselves could monitor and start live teleauscultation sessions with remote doctors.

Percussion might be needed in certain clinical scenarios, such as suspicion of vertebral fractures. Self-performed percussion is not feasible, but assisted percussion could be performed provided that the person performing the test is trained in where and how hard to percuss. Considering the aggressiveness of this maneuver and its potential implications for an augmentation of the pain, authors believe that percussion is best performed by a trained clinician, and therefore, it is not included among the list of assisted-physical examination tests.

### 3.7. Treatment planning

The planning of an analgesic treatment is the final step of a clinical encounter. Remote prescription of a drug requires careful explanation to the patient and safe delivery of such prescription to a pharmacy. Education about drug therapy or other crucial aspects of health matters related to chronic pain syndromes should not be particularly affected by conducting the visit remotely, whether by telephone or video. Clinicians should have access to a library of documents or websites with useful infographics or pamphlets that can be provided to patients remotely.

E-prescription programs vary widely within and between different countries and have been incorporated into clinical routines independently of TH approaches. When prescriptions must reach the patient's pharmacy of choice, they should be sent using secure methods to safeguard that it reaches the correct recipient while also maintaining the patients' details confidential. In the absence of sophisticated methods such as secure electronic health record platforms that can automatically send prescriptions to the patient's pharmacy of choice, secure e-mailing or faxing seem the most reliable methods.

Nonpharmacological medical treatments and indications for consultation with other clinical disciplines simply require an explanation of the purpose of the therapy offered and, when appropriate, obtaining patient consent which can only be verbal when conducting the visit remotely.

### 3.8. Patient considerations

Scheduled telephone calls with pain nurses were already a crucial part of routine clinical care for many years. Monitoring changes in symptom severity after initiation of opioids or opioid rotation, following interventional procedures, intravenous infusion, etc. were part of routine clinical calls. In addition, patients could make unscheduled phone calls to report changes of their symptoms or side effects which prevent them from attending emergency services. Patients had little difficulty in adapting to the new reality involving physicians conducting routine follow-up consultations. Their experience was rarely less than very positive because this system allowed patients direct access to their treating clinician and avoiding unnecessary travel to the clinic. In addition, the fact that clinicians were able to reach them more frequently was experienced as very positive.

As part of routine quality assurance evaluation, we investigated the implementation of the pediatric telemedicine program through use of an informal survey emailed to existing patients assessed through video between March to May 2020. We used the Patient Assessment of Communication During Telemedicine survey focused largely on empathy and perceived understanding of the therapeutic relationship.^21^ We also inquired about perceptions regarding the satisfaction with the pain management when assessed by TH compared with previous in-clinic visits. The survey was further stratified on questions regarding skills and competence, interpersonal skills, and quality of care received by telemedicine. Twenty-two percent (32/146) responded, with the majority being females (90%), of average age 15 years,^11–17^ and with the majority (81.3%) having pain primary pain conditions.

Patients perceived that the physician using telemedicine wanted to know about the condition, cared about their problems, and were comfortable discussing them with patients and parents and finally that they had a good understanding of the condition, its consequences, and the recommended management. Physicians were seen to ask more than yes or no questions, asked about expectations of care, were emotionally invested, included patients in discussions on care, listened to patients' ideas of their care, and tried to discover all problems with the patient. Patients believed that physicians were supportive partners, interested in what they were saying thus trusted. Physicians did not seem in a hurry, bored, or missed important information. They did not seem technically incompetent, ignoring medical problems, nervous, or condescending. Most patients would recommend telemedicine visits to others, and it was largely perceived positively. Patients liked that remote follow-up was convenient and satisfactory, yet they denied feeling more connected to their care because of telemedicine contact and did not believe their pain was better managed. Surprisingly, when asked separately to patients and parents whether they preferred in-person consultations, patients favored in person and their parents favored telemedicine. This may be due to the nature of the chronic pain condition, where patients may require multiple visits to a variety of multimodal treatment, including physiotherapy, psychology, and medical therapy, rather than a single visit with a physician. Moreover, chronic pediatric pain patients have been noted to develop strong emotional and psychological connections to their clinics, which were abruptly disrupted by COVID-19.

Further research is needed to identify which patients will benefit from telemedicine consults vs those who should be rather seen in person. Our findings agree with the published literature,^2^ where increased access to care with real-time virtual consultations in pediatric emergencies services^12^ and increased collaboration between patients and providers in general pediatric clinics^27^ have been demonstrated. Our research targeted for first-time chronic pediatric pain patients.

## 4. Telehealth for pain medicine, immediate future

Telemedicine for the care of chronic pain patients was available before the pandemic, but actual implementation was never pushed forward. The pandemic forced action. An important reflection must be undertaken to decide the future of the pain medicine specialty for the time when clinical activities return to normal. Should we return to the previous clinical model based exclusively on in-person consultations, or should we further integrate telemedicine into routine clinical practice?

The advantages of remote clinical care have been described in existing reviews. A summary from the authors' clinical experience about pros and cons of TH is presented in Table 3. We want to highlight that patients are in control of their care, their time is maximized at home, they are partners in their own pain care, and they are provided with direct means of communicating their pain syndromes. This responsibility comes with the possibility of miscommunication, “broken telephone,” and the lack of means to truly break a patient from their own environment; that is, they may be with their family, rather than alone in clinic, and thus may provide less honest answers to clinical inquiry. This latter concern may be offset by the comfort level patients report in their home, but this has yet to be formally investigated.

**Table 3:** Pros and cons of telemedicine for the patient and physician.

Summary	PROS	CONS
Patient considerations	Avoid travel to clinic Facilitates work/personal life reconciliation	Communication less effective Lack of paper interaction Upgrade tools for remote care* Internet connection† Lack of universal access, social inequalities
Administrative considerations	Facilitates teleworking Avoids physical interaction with challenging patients/families Improved time management E-mail communications easily documented	Update patient's details for remote contact Time consuming patient education about remote tools Requires a seamless electronic medical record software†
Health care system	Safe transportation cost to health care facilities Less need of dedicated spaces for patients If hospital closures, clinical care still provided	Upgrade spaces and tools for remote care* Internet connection, software licenses†
Clinical considerations	Allows teleworking if clinician cannot be physically present at the clinic	Potentially inaccurate clinical impression *(lack of body language, limited physical examination).* Weakens clinical alliance with patients Phone/IT systems dependent
Clinical training considerations	Facilitates remote clinical training	Manual skills cannot be perfectioned
Clinical research considerations	Allows remote communication between research team–participant Allows remote data collection	Phone/IT systems dependent Research methods requiring physical presence (clinical acts) are challenging

* One time only expense.

† Recurrent expenses.

Once experienced, it is hard not to acknowledge the advantages of telemedicine for chronic pain management. Although undoubtably inferior to an in-person clinical evaluation, remote consultations for chronic pain cases are surely preferred by those living far from the clinic and whose pain is well managed with the current analgesic therapy. For telemedicine to become a routine part of the specialty of pain medicine, changes are needed at various levels such as administrative, structural, financial, and clinical. There are also repercussions at the academic and research levels to consider.

Administrative changes will involve updating patients' profiles to allow faster and seamless communication by e-mail or telephone. E-mail should be adopted as a routine channel of communication between patients and front desks, but drawbacks such as delays in replies, potential breach of confidentiality, and identity thefts must be considered. For communications from or to patients that are considered urgent and/or very important, this system remains suboptimal.

Although the decision to perform a visit remotely remains a clinical decision, patients must have a say in this matter when clinically appropriate and technically feasible. Appointment lists will have to be built clustering the type of appointments, thus potentially liberating clinical areas and allowing teleworking.

Structural changes are required to optimize tele-care such as improvement of internet connections at health care institutions, updating computers' software, installing cameras, microphones and speakers, and updating the phone system for hands-free calls.

The care of the patient remains the top priority, even if conducted remotely. Remote clinical care abides by the same deontological responsibilities and professional rules set out by professional colleges and health authorities. It is reasonable to assume that clinicians' remuneration should remain equivalent to the quality of the care provided and not to the method chosen to deliver it.

Adaptation to telemedicine for clinicians is not particularly hard once the obvious limitations are acknowledged and technological barriers overcome. New routines will need to be adopted such as documenting consent for remote visits, building safe and effective methods to deliver the treatments, and to schedule further appointments. Efforts to remotely identify symptoms or signs suggesting disease progression or onset of worrisome clinical syndromes must be made and taught. Regulating remote prescriptions of pharmacological agents considered potentially dangerous, such as opioids, sedatives, and cannabinoids, will need to be updated.

Education in pain medicine relies fundamentally on the presence of patients and direct supervision of trainees by clinicians. Telemedicine definitively affects that in-person interaction. Training at simulation centers or advanced remote methods such as those set out in the next segment can facilitate clinical teaching, although they will never fully substitute for in-person care.^2,3^

Clinical research with remote protocols is a new reality nowadays. Internet-based data collection software and applications are routinely used to collect clinical data from patients participating in clinical research projects. The immediate future of clinical pain medicine research will need to adopt tools to facilitate that patients do not need to be present at all during the research conduction, such as virtual consent.^28^

## 5. Telehealth for chronic pain management, future directions

Telehealth consultation depends on the integration of the information provided by instruments and tools. A system that provides a better immersive experience coupled with real-time consultation could improve both the clinician's and patient's experience. Virtual reality uses 2-dimensional or 3-dimensional technology, allowing patients and providers to access and interact within a “virtual world.” Virtual reality requires multisensory input to create this world. Examples of virtual reality include augmented reality, modified reality, haptic sensation, high definition 3D (HD3D), 3D holograms, and the combination of several of these novel technologies.

High definition 3D telemedicine effect involves the rapid presentation of slightly different images to both eyes to generate the impression of depth and realism. The HD3D video consultation and/or diagnostic-focused interaction between patient and doctors enhances the telepresence sense of “being there.” Augmented reality involves projection of a virtual image being overlaid onto the physical world environments. The system enhances the “real-world” experience with images, videos, clinical data, instructions, etc. The perception of the real world of patients and providers remains intact, with a digital object inserted into their world.^20^ Augmented reality has been extensively used in physical rehabilitation. It has also shown promising results in behavioral therapy, with a focus on improving function and reducing distress in patients with chronic pain conditions.^32^

Using augmented reality, a mentor could provide consultation based on the 3D model and remote procedural training.^13^ The glass technology is a wearable computer that has the capability to add information to what the wearer sees. Integrated cameras can also provide a wearer's “viewpoint” to a remote device through live video streaming. Augmented reality glass has been used in telewound care and could also be used for some interventional and rehabilitation techniques in pain treatment.^38^

Currently, the physical examination during telemedicine interactions is mostly limited to what is visible through video. Physical examination requires a health care provider or a proxy to interact with the patient and report the findings. Augmented reality associated with haptic technologies could help to provide the element of touch lost during typical telemedicine consultations. Haptic technology devices allow the creation of an experience of touch by interfacing with users through force, vibration, or motion. These devices target the human cutaneous and kinesthetic sensory systems. Haptics technology allows for a remote observer to palpate, evaluate tenderness, range of motion, strength, muscular tone, and evaluate cutaneous sensation.^18^

The combination of augmented reality and haptic devices had a demonstrable positive effect on rehabilitation. Augmented reality systems allow visual monitoring of the patients' performance, and haptic machines can record quantitative information while they exercise. The same concept can be easily transferred to both the diagnostic and physical treatment of patients with chronic pain.^7^

Touchless ultrasonic haptic technology uses electronically controlled phased arrays of ultrasound transducers to create high acoustic pressure points in mid-air that can be felt with bare hands. It delivers advanced, multipoint mid-air tactile sensations directly onto users' hands and fingertips. This advanced technology allows dynamic real-time hand gesture interactions and holographic object manipulations.^8^

The next frontier could be represented by haptic bio-holograms. This represents the integration of modified reality, wearable biosensing, and mid-air haptics into an audio-visual and haptic-synchronized interaction with live holographic 3D objects. The holograms can be seen, “touched,” and felt and also behave (graphically and haptically) according to bio-sensed data.^22^

Chronic pain care can be conducted routinely by TH. With the advancement in technology and telecommunication networks, these new technologies and service models can streamline and automate critical aspects of the screening, monitoring, and treatment processes. Recent advances in artificial intelligence using deep learning have resulted in a paradigm shift in advanced medical care. Telemedicine platforms that can be coupled with automated algorithms incorporating artificial intelligence and big data analytics will further revolutionize the growing field of pain diagnosis and treatment.^26,36^

## Disclosures

Authors declare no conflict of interest related with the topic presented.

## References

[1] Adamse C Dekker-Van Weering MG van Etten-Jamaludin FS Stuiver MM. The effectiveness of exercise-based telemedicine on pain, physical activity and quality of life in the treatment of chronic pain: a systematic review. J Telemed Telecare 2018;24:511–26. 28696152 10.1177/1357633X17716576

[2] Agha Z Schapira RM Laud PW McNutt G Roter DL. Patient satisfaction with physician–patient communication during telemedicine. Telemed And E-Health 2009;15:830–9. 10.1089/tmj.2009.0030 19919189

[3] Bellido JC de Pietro G Sannino G. Proceedings of the 8th ACM Int. Conf. on Pervasive Technologies Related to Assistive Environments. 2015;30:1–8.

[4] Bennett RM Goldenberg DL. Fibromyalgia, myofascial pain, tender points and trigger points: splitting or lumping? Arthritis Res Ther 2011;13:117. 21722339 10.1186/ar3357 PMC3218900

[5] Bhimani RH Cross LJS Taylor BC Meis LA Fu SS Allen KD Krein SL Do T Kerns RD Burgess DJ. Taking ACTION to reduce pain: ACTION Study rationale, design and protocol of a randomized trial of a proactive phone-based coaching intervention for chronic musculoskeletal pain among african Americans. BMC Musculoskelet Disord 2017;18:15. 28086853 10.1186/s12891-016-1363-6 PMC5237146

[6] Blue R Yang AI Zhou C De Ravin E Teng CW Arguelles GR Huang V Wathen C Miranda SP Marcotte P Malhotra NR Welch WC K JY. Telemedicine in the era of coronavirus disease 2019 (COVID-19): a neurosurgical perspective. World Neurosurg 2020;139:549–57. 32426065 10.1016/j.wneu.2020.05.066 PMC7229725

[7] Borresen A Wolfe C Lin C-K Tian Y Raghuraman S Nahrstedt K Prabhakaran B Annaswamy T. Usability of an immersive augmented reality based telerehabilitation system with haptics (ARTESH) for synchronous remote musculoskeletal examination. Int J Telerehabil 2019;11:23–32. 31341544 10.5195/ijt.2019.6275 PMC6597147

[8] Broeren J Dixon M Sunnerhagen KS Rydmark M. Rehabilitation after stroke using virtual reality, haptics (force feedback) and telemedicine. Stud Health Technol Inform 2006;124:51–6. 17108503

[9] Craig JJ McConville JP Patterson VH Wootton R. Neurological examination is possible using telemedicine. J Telemed Telecare 1999;5:177–81. 10628033 10.1258/1357633991933594

[10] Eccleston C Blyth FM Dear BF Fisher EA Keefe FJ Lynch ME Palermo TM Reid MC Williams ACdeC. Managing patients with chronic pain during the COVID-19 outbreak: considerations for the rapid introduction of remotely supported (eHealth) pain management services. PAIN 2020;161:889–93. 32251203 10.1097/j.pain.0000000000001885 PMC7172975

[11] Ekeland AG Bowes A Flottorp S. Effectiveness of telemedicine: a systematic review of reviews. Int J Med Inform 2010;79:736–71. 20884286 10.1016/j.ijmedinf.2010.08.006

[12] Foster CC Macy ML Simon N-J Stephen R Lehnig K Bohling K Schinasi DA. Emergency care connect: extending pediatric emergency care expertise to general emergency departments through telemedicine. Acad Pediatr 2020;20:577–84. 32112864 10.1016/j.acap.2020.02.028

[13] Garrett B Taverner T McDade P. Virtual reality as an adjunct home therapy in chronic pain management: an exploratory Study. JMIR Med Inform 2017;5:e11. 28495661 10.2196/medinform.7271 PMC5445235

[14] Gogia SB Maeder A Mars M Hartvigsen G Basu A Abbott P. Unintended consequences of tele health and their possible solutions. Contribution of the IMIA Working Group on Telehealth. Yearb Med Inform 2016;1:41–6. doi: 10.15265/IY-2016-012. 27830229 PMC5171569

[15] Hailey D Roine R Ohinmaa A. Systematic review of evidence for the benefits of telemedicine. J Telemed Telecare 2002;8(suppl 1):1–30. 10.1258/1357633021937604 12020415

[16] Hanif Khan F Hanif R Tabassum R Qidwai W Nanji K. Patient Attitudes towards physician nonverbal behaviors during consultancy: result from a developing country. ISRN Fam Med 2014;2014:473654. 10.1155/2014/473654 PMC4041264 24977140

[17] Helstrom A Haratz J Chen S Benson A Streim J Oslin D. Phone-based management of chronic pain in older adults in an integrated care program. Int J Geriatr Psychiatry 2018;33:779–85. 29498774 10.1002/gps.4860

[18] Kaylor J Hooper V Wilson A Burkert R Lyda M Fletcher K Bowers E. Reliability testing of augmented reality glasses technology: establishing the evidence base for telewound care. J Wound Ostomy Continence Nurs 2019;46:485–90. 31633610 10.1097/WON.0000000000000585

[19] Lamb L Pereira JX Shir Y. Nurse case management program of chronic pain patients treated with methadone. Pain Manag Nurs 2007;8:130–8. 17723930 10.1016/j.pmn.2007.05.002

[20] Leronimakis KM Cain JA Switzer MS Odineal DD Deacy TK Stein MTO E Colombo R Colombo CJ. Leveraging tele-critical care capabilities for clinical trial consent. Crit Care Explor 2020;2:e0167. 32766563 10.1097/CCE.0000000000000167 PMC7339318

[21] Mani S Sharma S Ka Singh D. Concurrent validity and reliability of telerehabilitation-based physiotherapy assessment of cervical spine in adults with non-specific neck pain. J Telemed Telecare 2021;27:88–97. 31272309 10.1177/1357633X19861802

[22] Ochiai Y Hoshi T Rekimoto J. Three-dimensional mid-air acoustic manipulation by ultrasonic phased arrays [published correction appears in PLoS One. 2014;9(7):e102525]. PLoS One 2014;9:e97590. 24849371 10.1371/journal.pone.0097590 PMC4029622

[23] Ramelet A-S Fonjallaz B Rio L Zoni S Ballabeni P Rapin J Gueniat C Hofer M. Impact of a nurse led phone intervention on satisfaction and health outcomes of children with inflammatory rheumatic diseases and their families: a crossover randomized clinical trial. BMC Pediatr 2017;17:168. 28716081 10.1186/s12887-017-0926-5 PMC5513092

[24] Remy C Borniard J Perez J. Analysis of unscheduled phone calls received by a specialized cancer pain nurse. Pain Manag Nurs 2020;21:255–8. 31473170 10.1016/j.pmn.2019.07.009

[25] Richardson BR Truter P Blumke R Russell TG. Physiotherapy assessment and diagnosis of musculoskeletal disorders of the knee via telerehabilitation. J Telemed Telecare 2017;23:88–95. 26985005 10.1177/1357633X15627237

[26] Saad W Bennis M Chen M. A vision of 6G wireless systems: applications, trends, technologies, and open research problems. IEEE Network 2020;34:134–42.

[27] Sauers‐Ford HS Hamline MY Gosdin MM Kair LR Weinberg GM Marcin JP Rosenthal JL. Acceptability, usability, and effectiveness: a qualitative Study evaluating A pediatric telemedicine program. Acad Emerg Med 2019;26:1022–33. 30974004 10.1111/acem.13763 PMC6732030

[28] Shah S Diwan S Kohan L Rosenblum D Gharibo C Soin A Sulindro A Nguyen Q Provenzano DA. The technological impact of COVID-19 on the future of education and health care delivery. Pain Physician 2020;23:S367–80. 32942794

[29] Shanthanna H Cohen SP Strand N Lobo CA Eldabe S Bhatia A Narouze S. Recommendations on chronic pain practice during the COVID-19 pandemic. A Joint Statement by American Society of Regional Anesthesia and Pain Medicine (ASRA) and European Society of Regional Anesthesia and Pain Therapy (ESRA). 2020. Available at: https://www.asra.com/guidelines-articles/original-articles/covid-19-resources/covid-19-resources/legacy-b-blog-posts/2020/03/27/recommendations-on-chronic-pain-practice-during-the-covid-19-pandemic. Accessed March 3, 2021.

[30] Slattery BW Haugh S O'Connor L Francis K Dwyer CP O'Higgins S Egan J McGuire BE. An evaluation of the effectiveness of the modalities used to deliver electronic health interventions for chronic pain: systematic review with network meta-analysis. J Med Internet Res 2019;21:e11086. 31317869 10.2196/11086 PMC6668295

[31] Steele L Lade H McKenzie S Russell TG. Assessment and diagnosis of musculoskeletal shoulder disorders over the internet. Int J Telemed Appl 2012;2012:945745. 23193395 10.1155/2012/945745 PMC3501948

[32] Stranieri A Collmann R Borda A. High definition 3D telemedicine: the next frontier? Stud Health Technol Inform 2012;182:133–41. 23138088

[33] Suh S-R Lee MK. Effects of nurse-led phone-based supportive interventions for patients with cancer: a meta-analysis. Oncol Nurs Forum 2017;44:E168–84. 28632251 10.1188/17.ONF.E168-E184

[34] The Physician, Telemedicine and Information and Communications Technologies. Publication of the Collège des médecins du Québec, Canada. 2015. Available at: http://www.cmq.org/publications-pdf/p-1-2015-02-01-en-medecin-telemedecine-et-tic.pdf. Accessed March 3, 2021.

[35] Thompson JC Cichowski SB Rogers RG Qeadan F Zambrano J a Wenzl C Jeppson PC Dunivan GC Komesu YM. Outpatient visits versus phone interviews for postoperative care: a randomized controlled trial. Int Urogynecol J 2019;30:1639–46. 30783704 10.1007/s00192-019-03895-z PMC6699921

[36] Ting DS Gunasekeran DV Wickham L Wong TY. Next generation telemedicine platforms to screen and triage. Br J Ophthalmol 2020;104:299–300. 31796427 10.1136/bjophthalmol-2019-315066

[37] Tuckson RV Edmunds M Hodgkins ML. Telehealth. N Engl J Med 2017;377:1585–92. 29045204 10.1056/NEJMsr1503323

[38] Wang S Parsons M Stone-McLean J Rogers P Boyd S Hoover K Meruvia-Pastor O Gong M Smith A. Augmented reality as a telemedicine platform for remote procedural training. Sensors (Basel) 2017;17:2294. 10.3390/s17102294 PMC5676722 28994720

[39] Weinstein RS Krupinski EA Doarn CR. Clinical examination component of telemedicine, telehealth, mHealth, and connected health medical practices. Med Clin North Am 2018;102:533–44. 29650074 10.1016/j.mcna.2018.01.002

